# The struggle to find reliable results in exome sequencing data: filtering out Mendelian errors

**DOI:** 10.3389/fgene.2014.00016

**Published:** 2014-02-12

**Authors:** Zubin H. Patel, Leah C. Kottyan, Sara Lazaro, Marc S. Williams, David H. Ledbetter, hbGerard Tromp, Andrew Rupert, Mojtaba Kohram, Michael Wagner, Ammar Husami, Yaping Qian, C. Alexander Valencia, Kejian Zhang, Margaret K. Hostetter, John B. Harley, Kenneth M. Kaufman

**Affiliations:** ^1^Division of Rheumatology, Center for Autoimmune Genomics and Etiology, Cincinnati Children's Hospital Medical Center, CincinnatiOH, USA; ^2^Medical Scientist Training Program, University of Cincinnati College of Medicine, CincinnatiOH, USA; ^3^Department of Veterans Affairs, Veterans Affairs Medical Center – Cincinnati, CincinnatiOH, USA; ^4^Genomic Medicine Institute, Geisinger Health System, DanvillePA, USA; ^5^Division of Biomedical Informatics, Cincinnati Children's Hospital Medical Center, CincinnatiOH, USA; ^6^Division of Human Genetics, Cincinnati Children's Hospital Medical Center, CincinnatiOH, USA; ^7^Division of Infectious Disease, Cincinnati Children's Hospital Medical Center, CincinnatiOH, USA

**Keywords:** whole exome sequencing, variant filtering, next-generation sequencing, disease causative polymorphisms, Mendelian errors, Mendel errors, CASSI

## Abstract

Next Generation Sequencing studies generate a large quantity of genetic data in a relatively cost and time efficient manner and provide an unprecedented opportunity to identify candidate causative variants that lead to disease phenotypes. A challenge to these studies is the generation of sequencing artifacts by current technologies. To identify and characterize the properties that distinguish false positive variants from true variants, we sequenced a child and both parents (one trio) using DNA isolated from three sources (blood, buccal cells, and saliva). The trio strategy allowed us to identify variants in the proband that could not have been inherited from the parents (Mendelian errors) and would most likely indicate sequencing artifacts. Quality control measurements were examined and three measurements were found to identify the greatest number of Mendelian errors. These included read depth, genotype quality score, and alternate allele ratio. Filtering the variants on these measurements removed ~95% of the Mendelian errors while retaining 80% of the called variants. These filters were applied independently. After filtering, the concordance between identical samples isolated from different sources was 99.99% as compared to 87% before filtering. This high concordance suggests that different sources of DNA can be used in trio studies without affecting the ability to identify causative polymorphisms. To facilitate analysis of next generation sequencing data, we developed the Cincinnati Analytical Suite for Sequencing Informatics (CASSI) to store sequencing files, metadata (eg. relatedness information), file versioning, data filtering, variant annotation, and identify candidate causative polymorphisms that follow either *de novo*, rare recessive homozygous or compound heterozygous inheritance models. We conclude the data cleaning process improves the signal to noise ratio in terms of variants and facilitates the identification of candidate disease causative polymorphisms.

## INTRODUCTION

Next-generation sequencing (NGS) has emerged as a powerful tool to investigate the genetic etiology of diseases. The use of NGS data has revolutionized clinical treatment and bench research. In general, the data generated in a NGS study are massive by comparison to that generated by a genome-wide genotyping array. In NGS, a fastq file of millions of short DNA sequences is generated for each sample. These fastq files are aligned to the reference genome using one of many different alignment tools. The alignment programs create a sequence alignment/map (SAM file) or a binary alignment/map (BAM file; Yu and Sun, 2013). It is widely appreciated that NGS generates a large number of sequencing errors. The extraordinary quantity of data generated even with a low error rate generates a large number of sequencing artifacts which will likely be called variants. This gives the appearance that NGS does not compare well with Sanger sequencing or genotyping arrays (4), but we show herein that the error rate of NGS of the called variants can be substantially reduced with the relative preservation of the vast majority of the data. To address the limitations imposed upon NGS studies by sequencing artifacts, we find refuge in redundancy. Typically, researchers obtain 40–200 reads of each base. Therefore, SAM and BAM files are large files and contain hundreds of millions of short sequences aligned to the reference genome. Variant callers such as the Genome Analysis Tool Kit (GATK) are used to generate a list of the variants in the variant call format (VCF; McKenna et al., 2010). VCF files contain meta-information for each variant relative to a known reference genome sequence, as well as quality measurements for each subject’s individual genotypes. These individual quality metrics include the overall number of reads at each position as well as the number and depth of alleles detected. In addition to predicting the nucleotide base or generating a base call calculated from a statistical algorithm, GATK also calculates a confidence score for the predicted nucleotide, the genotype quality score (Nielsen et al., 2011).

A multi-sample VCF file includes all of the genotypes for which at least one subject has a variant. Due to the flexibility of the format, the information contained in these files can vary widely. Furthermore, different variant callers are known to produce different calls (Rosenfeld et al., 2012; Liu et al., 2013). To further complicate matters, there is no currently agreed upon consensus to guide the analytical choices that are made when deciding which variant calls to include in a VCF file [as reviewed in Nekrutenko and Taylor (2012)]. One approach is to exclude (or filter) variants from a VCF file based on various criteria. These filters are based on a meta- or individual-sequencing parameter used to remove a particular variant. For example, variants can be filtered based upon the read depth (the number of the times the variant was detected), ratio of reads that contained the reference and alternate genotype calls (alt read), or by genotype quality scores. A recent comparison of the most common next-generation sequencing platforms and methodologies demonstrated that only 57% of the variants are common amongst five different pipelines using the same initial data (O’Rawe et al., 2013).

Typically a whole exome NGS experiment will generate ~50–70 million bases of sequence. Greater than 99.99% of the bases match the reference genome. The remaining 0.01% of bases that differ from the reference genome is identified as variants. Importantly, most sequencing artifacts do not match the reference genome and are mis-identified as variants. Thus, identifying variants also has the effect of concentrating the sequencing artifacts. These sequence artifacts can be detected by identifying non-concordance of sequence from multiple assays of the same samples or as Mendelian errors if family data are available. Mendelian errors are genotypes that are found in the child that could not have been inherited from either parent.

After obtaining the data from a whole exome NGS experiment, analytical strategies range from identifying novel variants, to performing genetic association studies, to identifying variants that are candidates for potentially causing disease. Within the last 5 years, exome sequencing methods have been employed to successfully identify mutations in novel genes for a number of genetic conditions, including Sensenbrenner syndrome, Kabuki syndrome, and Miller syndrome (Gilissen et al., 2010; Ng et al., 2010a,b). One highly successful strategy uses the healthy parents of a patient with a severe disease to identify genetic variants in the patient that were not inherited, termed *de novo* variants. In fact, disruptive *de novo* variants appear to cause a substantial proportion of intellectual disability and many rare genetic disorders (Hoischen et al., 2010, 2011; Vissers et al., 2010; Bartnik et al., 2011; Filges et al., 2011; Gilman et al., 2011; Girard et al., 2011; Gonzalez-del Pozo et al., 2011; Paulussen et al., 2011; Xu et al., 2011; Bujakowska et al., 2012; Dauber et al., 2012; Harakalova et al., 2012; Iossifov et al., 2012; Lederer et al., 2012; Lin et al., 2012; Neale et al., 2012; Need et al., 2012; Neveling et al., 2012; O’Roak et al., 2012b; Riviere et al., 2012; Sanders et al., 2012; Santen et al., 2012; Schrier et al., 2012; Tsurusaki et al., 2012; Van Houdt et al., 2012; Whalen et al., 2012).

Using a trio study design (father, mother, and child) we can identify non-inherited variants in a child. These variants are sequencing errors, somatic mutations, or *de novo* mutations. We have used this analysis of trios as an opportunity to identify methodologies to filter the data to remove sequencing artifacts while retaining true mutations. In this study, we systematically assessed quality metrics to minimize Mendelian errors and identified a set of filters that remove these erroneous variants. These critical metrics are the depth of read (DP), the genotype quality score (GQ), and the alternate allele ratio. Filters based on these metrics were applied to a trio in which each family member was sequenced using three different sources of DNA (blood, saliva, and buccal cells). We tested the efficiency and specificity of our filters to remove sequencing artifacts by measuring the number of Mendelian errors and total variants removed by each filter singly and in combination. After testing the efficiency of our variant calling filters, we evaluated the filters on the concordance rate between identical samples from different DNA tissue sources. In order to make these analyses accessible to clinician researchers with limited command line programming experience, we have developed the Cincinnati Analytical Suite for Sequencing Informatics (CASSI) to seamlessly integrate the data storage, versioning, filtering, and annotation of NGS data through a web-based interface.

## METHODS

### DATA DESCRIPTION

We performed whole exome sequencing on a family trio. For this trio, three sources of DNA were obtained: blood, buccal, and saliva. Blood samples were collected from the three individuals using EDTA Vacutainer^TM^ Tubes (BD Franklin Lakes, New Jersey, USA). The buccal cells were collected by taking a cheek swab of each individual using the OGR-575 tubes from DNA-Genotek (Kanata, ON, Canada) and the saliva samples were collected by having each individual directly spit into the OGR-500 tube from DNA-Genotek. DNA was extracted using the DNeasy Blood and Tissue kit from Qiagen (Valencia, CA, USA). Each subject gave informed consent or assent approved by the institutional review board at Cincinnati Children’s Hospital Medical Center. We studied all samples by exome capture using the Illumina HiSeq 2000 100-base pair-end platform with the IlluminaTruSeq kit. (San Diego, CA, USA; In our experience, exome data generated with the AgilentSureSelect capture kit behaves similar to the data presented in this paper). Samples were sequenced at Perkin Elmer (Branford, CT, USA). These filters have been applied to data generated with IlluminaTrueSeq and AgilentSureSelect capture technologies.

Reads were aligned to the UCSC reference human genome assembly 37.68^1^ using BWA with the following commands: aln-o 1-e 10-i 5-k 2-l 32-t 4 (Li and Durbin, 2010). The mapping files in SAM format were converted to the BAM format using SAM tools version 0.1.19 (Li et al., 2009). The variants were called with the Broad Institute’s Genome Analysis Tool Kit (McKenna et al., 2010; DePristo et al., 2011) using the following commands: -T Unified Genotyper-dcov 1000-stand_call_conf 30.0-stand_emit_conf 30.0 – min_base_quality_score 20 -A Depth Of Coverage -A IndelType -A QualByDepth -A ReadPosRankSumTest -A FisherStrand -A MappingQualityRankSumTest -l INFO -glm.

We obtained an average of 94.5 million reads (range 80–115 million reads per subject, with 106-fold mean depth in the target regions). On average, approximately 98% of these reads were mapped to the human reference genome.

### DATA ANALYSIS

The VCF file generated by GATK was analyzed using Golden Helix Software (ver. 7.7.8) (Bozeman, MT, USA) and the newly developed CASSI. Variants located on the X and Y chromosomes were excluded from this analysis due to limitations in the Golden helix software. Only informative genotypes for each family were considered (genotypes where all three members of the trio were homozygous and identical to the reference sequence were removed). When sequencing data from multiple DNA sources were compared, only informative SNPs within the trio from one particular DNA source were included in the analysis. Variants were only removed based on individual quality measurements. When assessing the number of variants present in the child, all variants that remained in the child’s dataset after the filters were applied were counted (i.e., if the genotypes for both parents were removed with a filter, but the child’s genotype remained, this variant was still counted for the child), Mendelian errors were calculated for each variant by determining genotypes in the child which could not be inherited from the parents based on the parent genotypes. Mendelian errors were inferred for variants with a missing parental genotype if one parent and the child had opposite homozygous genotypes. The Mendelian error calculation did not include cases in which the child was heterozygous and only one parent was called. People interested in using CASSI should contact the corresponding author.

### THE CINCINNATI ANALYTICAL SUITE FOR SEQUENCING INFORMATICS

Cincinnati Analytical Suite for Sequencing Informatics was developed to address the data management requirements of next-generation sequencing data and to facilitate access to state-of-the-art open source analysis packages through a centralized web-based interface. CASSI analysis pipelines are run on the CCHMC 700 core Linux-based computational cluster and can also run on a local Linux-based machine. It leverages existing open source VCF file parsers and annotation tools including VCF tools, ANNOVAR, UCSC Genome Browser, Exome Variant Server, and dbGAP (Mailman et al., 2007; Wang et al., 2010; Danecek et al., 2011; Meyer et al., 2013).

Cincinnati Analytical Suite for Sequencing Informatics consists of a web-based front end driven by a MySQL backend. Users are able to upload their NGS data in the form of VCF files along with files that contain the family relatedness information for each sample (fam files). CASSI performs basic quality control checks on the uploaded files before they are accepted into the database. These checks include looking for an abundance of Mendelian errors and verifying the sex of the uploaded samples.

Fields that are commonly queried, such as sample name, family ID, and variant position are parsed out of the VCF file and indexed in the MySQL database. Storing only commonly queried fields in the database while keeping the genotype information in the original VCF file keeps the database size to a minimum while allowing quick access to the original VCF file and sample information. Analysis begins by sample selection and analysis type selection from the CASSI web interface. Using this information, CASSI then dynamically generates a custom pipeline for the specific type of analysis, which is launched using the LONI pipeline software (Rex et al., 2003; Dinov et al., 2010). Pipeline parameters can be changed through the LONI pipeline’s point-and-click interface. This allows for a seamless transition between search and analysis interfaces without requiring the user to have programmatic experience.

The LONI Pipeline simplifies computational cluster workflow creation using a drag and drop interface. CASSI users can launch and modify existing processing workflows directly from their web browser by using Java Web Start technology. The selected pipeline is preloaded into the LONI Pipeline client along with any sample data retrieved from the web interface. This is achieved by injecting the file locations of the sample data into a template .pipe LONI Pipeline file. Users are then free to modify the workflow. Input parameters are easily modified via the modules within the LONI Pipeline. The LONI Pipeline server interfaces with existing high performance computing environments in order to handle task dependencies and parallelization. In our case it communicates with the LSF job scheduler, but can also be used to communicate with other scheduling systems such as Oracle Grid Engine. Each modified LONI workflow can then be saved as XML and versioned using existing source control solutions (Subversion, Git, CVS, etc.). These XML files can be saved, shared, and submitted directly to a Linux-based machine.

For trio analysis, each member of the trio is initially extracted into a separate VCF file using VCFtools and then filtered on parameters selected by the user. After filtering for high quality variants, the samples are then scanned for amino acid altering variants (non-synonymous, splicing, insertions, deletions, and variations that alter initiation codons or stop codons) using the UCSC genome browser build 37 human Reference Sequence Gene table. Rare and novel variants are identified by filtering against the 1000 genomes project phase 1 v3 database^2^ and the NHLBI exome sequence project ESP6500 variant frequency data^3^. We also generated and use an internal allele frequency table of 312 whole exomes analyzed at CCHMC.

Individual and summary reports are generated for all candidate causative variants. These variants are annotated with chromosome, position, minor allele frequency, Gene name (hyperlinked to www.Genecards.org), transcript, and protein ID, amino-acid position and functional predictions based on dbSNP functional predictions Version 2 table.

### IDENTIFICATION OF POTENTIALLY CAUSATIVE MUTATIONS

Three different models of inheritance were used to identify candidate causative variants. We defined *de novo* variants as non-synonymous polymorphisms in which both parents are homozygous for the reference allele and the proband contained a heterozygous genotype. For homozygous recessive variants we required both parents to be heterozygous for the variant and the proband to be homozygous for the non-synonymous rare allele. For compound heterozygous polymorphism, we required the proband to contain at least two heterozygous non-synonymous polymorphisms in the same gene and neither parent could contain both variants. One variant could have a minor allele frequency in the general population up to 5% (based on the 1000 genome and exome sequence project); however, the other polymorphism had to have a minor allele frequency below 1%.

## RESULTS

We collected biological samples from the blood, saliva, and buccal cells of a child and the two biological parents. By extracting DNA from these nine samples and sequencing the exome, we used Mendelian errors to identify those variants that were most likely to be sequencing artifacts. In developing informatics filters for the NGS exome data, we aimed to retain the largest number of total variants while removing the largest possible number of Mendelian errors in the child.

**FIGURE 1:**
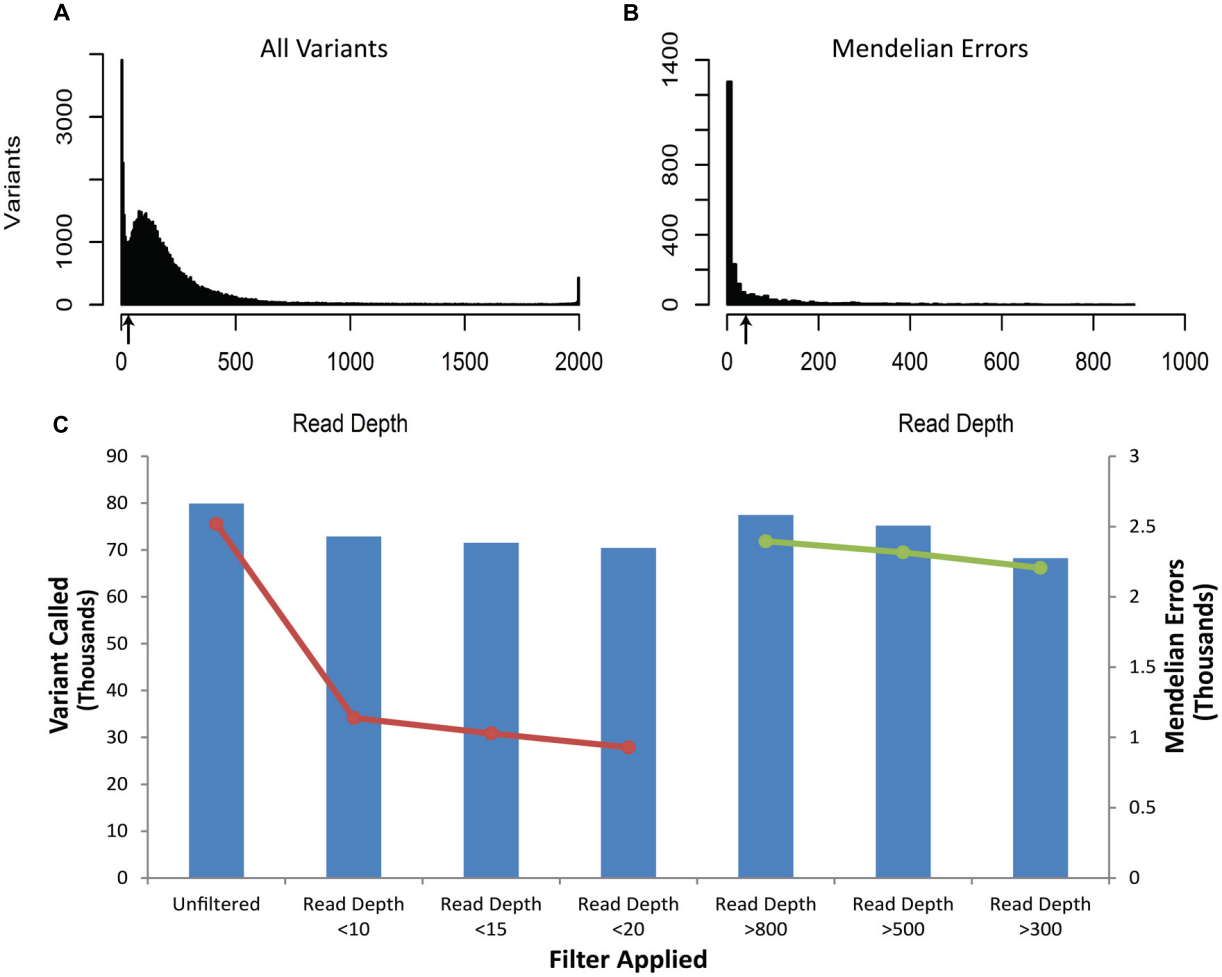
**Depth of Coverage:** The histograms depict the read depth by all called variants **(A)** and for the Mendelian errors **(B)** in the child. Similar histograms were obtained for the other samples regardless of the DNA source. The arrows depict the coverage depth cutoff (Depth < 15 reads) used to remove sequencing artifacts from the data. The bar graph depicts the number of variants remaining after applying an increasingly stringent read depth filter **(C)**. The line graph (-•-) depicts the number of Mendelian errors remaining after applying an increasingly stringent read depth filter **(C)**. The sequencing data of the DNA extracted from blood are shown and are representative of the other two DNA sources.

The vast majority of Mendelian errors in the unfiltered NGS data is due to sequencing error rather than *de novo* mutations based on the high fidelity of DNA replication in humans (Schmitt et al., 2009; Korona et al., 2011) and provide a method of tracking the effect of filters on sequencing artifacts. The initial analysis of the VCF file from the DNA obtained from blood revealed 2519 Mendelian errors compared to 79,911 called variants (3.15%). (These sequencing reads were mapped to 50 million bases, and for more than 99% of these calls, each of the subjects were homozygous for the reference base.) The mapped sequencing reads from different DNA samples of the same trio showed similar sequencing quality parameters (**Table 1**), similar proportion of Mendelian errors(3.05–3.15%), and total number of variants (79,234–79,911) called. We systematically applied filters to the VCF files until we identified the most efficient way to remove erroneous genotype calls while retaining the greatest number of true genotype calls. The first filter was based on the read depth (DP-number of sequencing reads that contain the variant) called within the trio (**Figure 1**). The read depth histogram of all variants in the proband using DNA isolated from blood shows a left-skewed distribution with two peaks located approximately at 5 reads and at 80 reads (near the mean read depth for this sample). A histogram for the same sample for the Mendelian errors shows the majority have a read depth below 12 reads and a sharp drop in the number of Mendelian errors as the read depth increases. Based on these results, we created filters with increasing stringency with a goal of removing the largest portion of the Mendelian errors while retaining the most variant calls. When applied to the unfiltered data a Read Depth < 10 removed 55% of the Mendelian errors, while retaining 92% of the called variants. With a Read Depth < 15 we were able to remove 59.2% of the Mendelian errors while retaining 90% of the called variants. Increasing the Read Depth filter above 15 had little effect on the number of Mendelian Errors removed (**Figure 1C**). Similar results were obtained with DNA isolated from buccal cells (61.6%) and saliva (56.1%).

**Table 1:** Sequencing quality parameters for all three individuals in blood, buccal, and saliva trio.

Sample	Percentage of reads with GQS > 30 (%)	Mean GQS	Percentage of targeted sequence covered (%)	Mean read depth
Blood – proband	84.19	33.46	97.07	167
Blood – father	83.58	33.25	96.46	146
Blood – mother	84.57	33.57	94.69	150
Buccal– proband	84.12	33.4	95.89	90
Buccal – father	84.82	33.63	96.73	155
Buccal – mother	85.04	33.71	97.35	136
Saliva – proband	83.38	33.17	95.95	106
Saliva – father	84.21	33.46	95.93	154
Saliva – mother	84.31	33.48	96.3	132

There were a number of variants called with a read depth > 2000. It is possible that the sequences for these variants are the result of a PCR artifact during library construction or corresponds to repetitive regions of the genome. We assessed filters that excluded variants with Read-Depth > 800, >500, and >300. After applying these filters, we removed 5, 8, and 12% of Mendelian errors and 3, 6, and 15% of the total variants, respectively. These data suggested that by filtering out variants with a large relative mean number of reads we were not specifically filtering out Mendelian errors, rather we were randomly removing Mendelian errors by decreasing the number of variants. Thus, we did not exclude variants with a relatively large read depth.**

**FIGURE 2:**
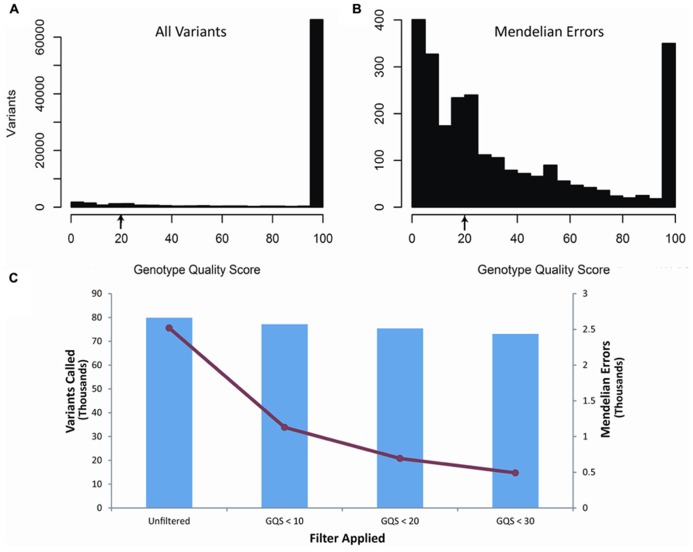
**Genotype Quality Score:** The histograms depict the distribution of genotype quality scores by all called variants **(A)** and by all the Mendelian errors **(B)** in the child. The arrows depict the genotype quality score cutoff (GQS < 20) used to remove sequencing artifacts from the data. The bar graph depicts the number of variants remaining after applying an increasingly stringent genotype quality score filter **(C)**. The line graph (-•-) depicts the number of Mendelian errors remaining after increasingly stringent filters are applied **(C)**. The sequencing data of the DNA extracted from blood are shown and are representative of the other two DNA sources.

Our second filter was based on the genotype quality score (GQ) of each of the variants called within the trio (**Figure 2**). The genotype quality score assesses the quality of sequencing information at each of the bases and ranges from 0 to 99 (see also discussion). A genotype quality score histogram for all variants found in the child blood DNA showed a right-skewed distribution with nearly all variants having GQ > 95. A similar histogram for the Mendelian errors shows a bi-modal distribution with a large portion of the data with a GQ < 20. Based on these results, we developed filters using increasingly stringent criteria and determined the effects of those filters on the number of Mendelian errors and the number of variants. The GQ < 20 filter removed 72.4% of the Mendelian errors while retaining 94% of the variants. These data suggest that the GQ filter is very selective and effective at removing Mendelian errors (**Figure 2**). This filter removed 70.8% of the Mendelian errors in the DNA isolated from buccal cells and 70.1% from saliva.

**FIGURE 3:**
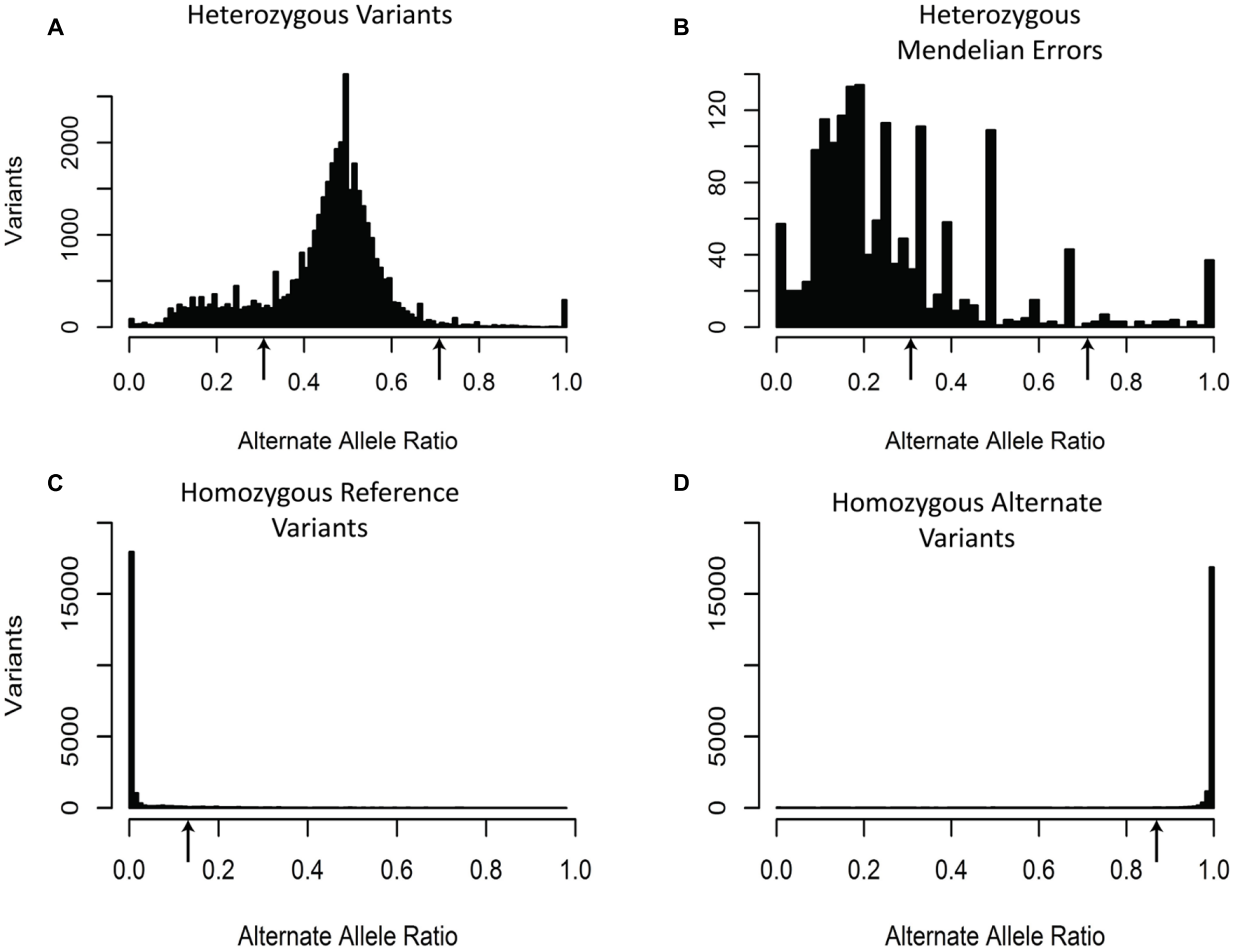
**Alternate Allele Ratio:** The histograms depict the distribution of Alternate Allele Ratio by all called variants with a heterozygous genotype **(A)**, by all the Mendelian errors with a heterozygous genotype **(B)**, by all the called variants with a homozygous reference genotype **(C)**, and by all the called variants with a homozygous alternate genotype **(D)**. The arrows depict the alternate allele ratios used to remove sequencing artifacts for heterozygous genotype calls (Alt-Allele Ratio > 0.7 or Alt-Allele Ratio < 0.3), homozygous reference genotype calls (Alt-Allele Ratio > 0.15), and homozygous alternate genotype calls (Alt-Allele Ratio < 0.85). The sequencing data of the DNA extracted from blood are shown and are representative of the other two DNA sources.

The third filter was based upon the expected alternate allele ratio (alt ratio) for a particular genotype (**Figure 3**). Variants are determined to be homozygous reference, heterozygous, or homozygous non-reference based upon algorithms in the caller. The alternate allele ratio is the proportion of the number of reads with the alternate allele at a position relative to the total number of reads at that same position. We use this metric to identify genotypes that are unlikely to be accurate given the available allele read depth. The histogram for variants with a heterozygous genotype displayed a distribution centered on 0.5. Interestingly, the heterozygous genotypes generated a peak in the histogram near 0.2 and often a smaller peak near 0.8. One possible explanation for these peaks is that the misalignment of two or more regions of the genome that are nearly identical but unevenly sequenced generate these ratios (**Figure 3**). As expected, the histogram for variants with a homozygous genotype for the reference allele showed a left-skewed distribution and the histogram for variants with a homozygous genotype for the alternate allele had a right-skewed distribution (**Figure 3**). Unlike the previous two filters, which used the same criteria for all the variants, the alt ratio filter has different selection criteria based upon the genotype of the sample for each variant. For this particular filter, all homozygous reference variants with alt ratio >0.15 were removed, all homozygous alternate variants with alt ratio <0.85 were removed, and all heterozygous variants with alt ratio <0.3 or alt ratio >0.7 were removed. With this filter, we were able to remove 61.8% of the Mendelian errors while retaining 88% of the total variants. In buccal cell DNA 57.7% of the Mendelian errors were removed and 61.5% in saliva.

**FIGURE 4:**
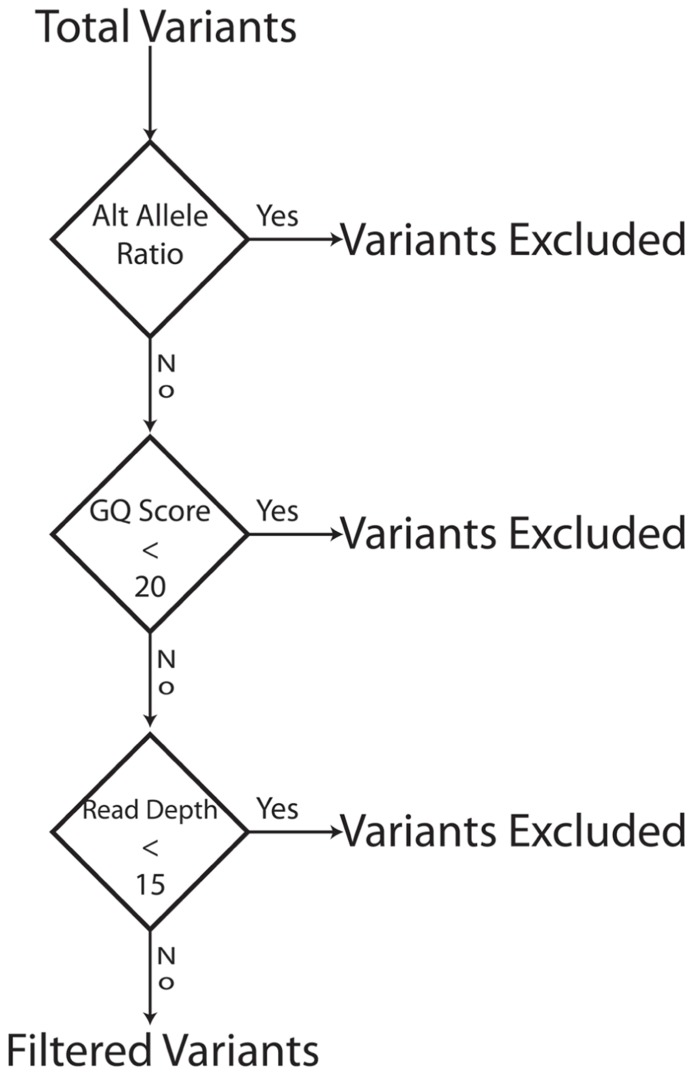
**Filter Schema:** The bioinformatics filters are sequentially applied. Variants that fail a filter are excluded and the next filter in the sequence is applied to the variants that pass a filter. To be excluded a variant has to fail only one filter. The order of filter is not important.

**FIGURE 5:**
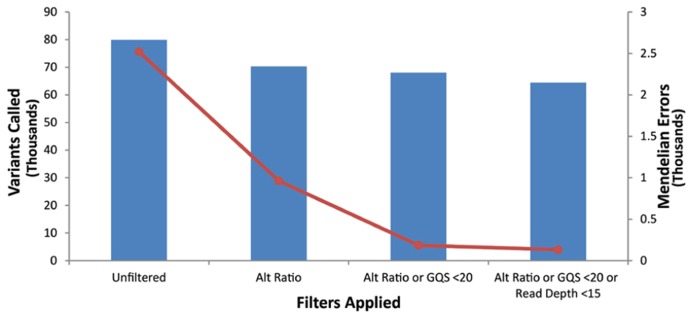
**Effect of applying multiple filters:** The bar-graph depicts the number of variants remaining after the application of filters on the data. The line-graph depicts the number of Mendelian Errors remaining after the application of additional filters (Top Panel). The sequencing data of the DNA extracted from blood is shown.

**FIGURE 6:**
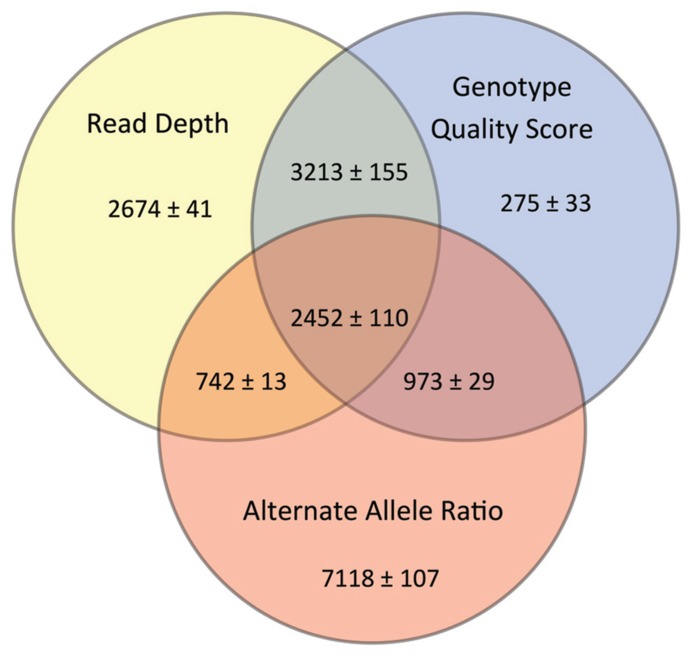
**Impact of Filters upon Data Quality:** The Venn diagram shows the number of variants excluded by each of the filters. The numbers represent a mean ± range/2 from all three DNA sources.

Our goal was to use multiple low stringency filters to selectively remove Mendelian errors while maintaining as much data as possible. Each of our filters based on the mean number of reads, genotype quality score, or alternate allele ratio was able to remove over half of the Mendelian errors in all of the DNA sources tested (Total Mendelian Errors: 2430–2519) while retaining a majority of the called variants (~90%). We determined the cut-off for each filter based on the variant and Mendelian Error histograms for each parameter (**Figures 1–3**) and a cost-benefit analysis setting the filter at the point at which increasing the filter stringency removed the same proportion of total variants as Mendelian errors. To improve the filtering, we sequentially applied these filters to our trio data (**Figure 4**). As mentioned previously, we were able to exclude 61.8% of the Mendelian errors while retaining 88% of the data by excluding variants with alternate allele ratios differing by 0.2 or greater from the expected alternate allele ratio (**Figure 3**). By adding a filter that also excluded variants with GQ < 20, we were able to exclude 92.7% of the Mendelian errors while retaining 85% of the original sequencing data in the blood sample (**Figure 5**). By excluding variants with read depth less than 15, we were able to further remove 50 Mendelian Errors. Although this may not seem to be a large decrease in the number of Mendelian Errors, these 50 Mendelian Errors comprise approximately 30% of the Mendelian Errors remaining after the Genotype Quality Score and the Alternate Allele Ratio filter are applied. By combining the three filters, we were able to remove 95% of the Mendelian errors, while retaining nearly 80% of the called variants. As shown in **Figure 6**, nearly 60% of the excluded variants are removed by only one filter, supporting our strategy of using multiple low-stringency filters to remove sequencing artifacts.

The Mendelian Error rate in unfiltered data is 3.7%. Based on the observation that the true error rate is three to four times the Mendelian Error rate detected by SNPs (Gordon et al., 1999) we estimate the actual error rate to be 9–10% in unfiltered NGS data. This estimate is in agreement with concordance rates seen when DNA from three different sources: blood, buccal-cells, and saliva for the same sample were compared. We assessed the concordance rates of non-filtered variants that were found in all of the DNA sources and found ~96% concordance for variants which were present in all three DNA sources (**Table 2**). The concordance dropped to ~84% if we also considered genotypes which were non-reference in one DNA source, but not called in one of the other two as being discordant. These unique genotypes were probably enriched for sequencing errors, as the vast majority were removed after applying the three filters described above (**Table 3**). After applying the filters, we were able to increase this concordance to greater than 99.999% amongst the variants that were common between the DNA sources (**Table 2**).

**Table 2:** Concordance analysis of DNA from three individuals was collected from three biological sources and sequenced.

DNA source	Sample	Concordance rate no filter applied (%)	Concordance rate all filters applied, not including variants that are unique to a single DNA source (%)	Concordance rate all filters applied, including variants that are unique to a single DNA source (%)
Blood vs. buccal	Individual 1	96.23	99.99	84.06
	Individual 2	96.61	99.99	84.71
	Individual 3	96.62	99.98	84.48
Blood vs. saliva	Individual 1	96.30	99.99	84.22
	Individual 2	96.53	99.99	84.42
	Individual 3	96.50	99.99	83.79
Buccal vs. saliva	Individual 1	96.27	99.99	84.14
	Individual 2	96.86	99.98	85.05
	Individual 3	96.73	99.99	84.22

*Using variants that were common amongst all of the DNA sources, we assessed the genotype concordance. We observed a significant improvement in the concordance after applying the three bioinformatics filters.*

**Table 3:** DNA from the proband (child) was collected from three biological sources and sequenced.

DNA source		Unique compared to blood	Unique compared to buccal	Unique compared to saliva	Unique compared to the other two sources
Blood	Unfiltered		2636	1997	1095
	Filtered		10	4	2
Buccal	Unfiltered	1267		1437	535
	Filtered	0		2	0
Saliva	Unfiltered	1268	1438		669
	Filtered	1	0		0

*The data set from each DNA source had unique variants that were not found in one or both of the other sources. After three bioinformatics filters were applied to the genotyping data, the number of unique genotypes was considerably reduced. The number of variants in one DNA source that are unique compared to another DNA source (e.g., blood compared to buccal) is different than the inverse comparison (e.g., buccal compared to blood).*

A trio study design is often used to identify candidate causative rare variants. In order to identify those amino-acid changing variants most likely to contribute functionally to a phenotype, we performed analyses to identify *de novo*, recessive homozygous (with less than 1% allele frequency in public sequencing databases), and compound heterozygous mutations. Filtering the sequencing data before this functional analysis reduced the apparent *de novo* mutations from 321 to 1. Similarly, potentially causal recessive homozygous variants were reduced from 32 to 3 and potentially causal compound heterozygous variants were reduced from 242 to 47. When these analyses were applied to each of the three DNA sources, we further reduced the number of potential causal variants to 0 apparent *de novo*, 3 rare homozygous, and 17 compound heterozygous variants which are identified in all three samples from different DNA sources (**Table 4**).

**Table 4:** Candidate causative sequence variants were identified in unfiltered and filtered data from the same trio that was sequenced three times using different DNA sources.

DNA source	De novo variants			Recessive homozygous variants			Compound heterozygous		
	Called	Unique to a single DNA source	Common to all DNA sources	Called	Unique to a single DNA source	Common to all DNA Sources	Called	Unique to a single DNA source	Common to all DNA sources
**Unfiltered**
Blood	321	228	12	32	3	23	242	47	153
Buccal	306	219	12	36	12	23	285	79	153
Saliva	304	230	12	28	4	23	284	80	153
**Filtered**
Blood	1	0	0	3	0	3	47	21	17
Buccal	0	0	0	3	0	3	39	11	17
Saliva	1	0	0	3	0	3	45	16	17

*Only novel or rare amino acid altering variants were considered. The numbers of unique variants (not found in any other DNA source) are indicated. Common variants were found in all three DNA sources. Variants found in two of the DNA sources, but not in the third are not included in this table. Filters applied to the variants included read depth >15, genotype quality score >20 and alternate allele ratio less than 0.15 for all homozygous reference, greater than 0.85 for homozygous alternate allele and between 0.3 and 0.7 for heterozygous genotypes.*

**FIGURE 7:**
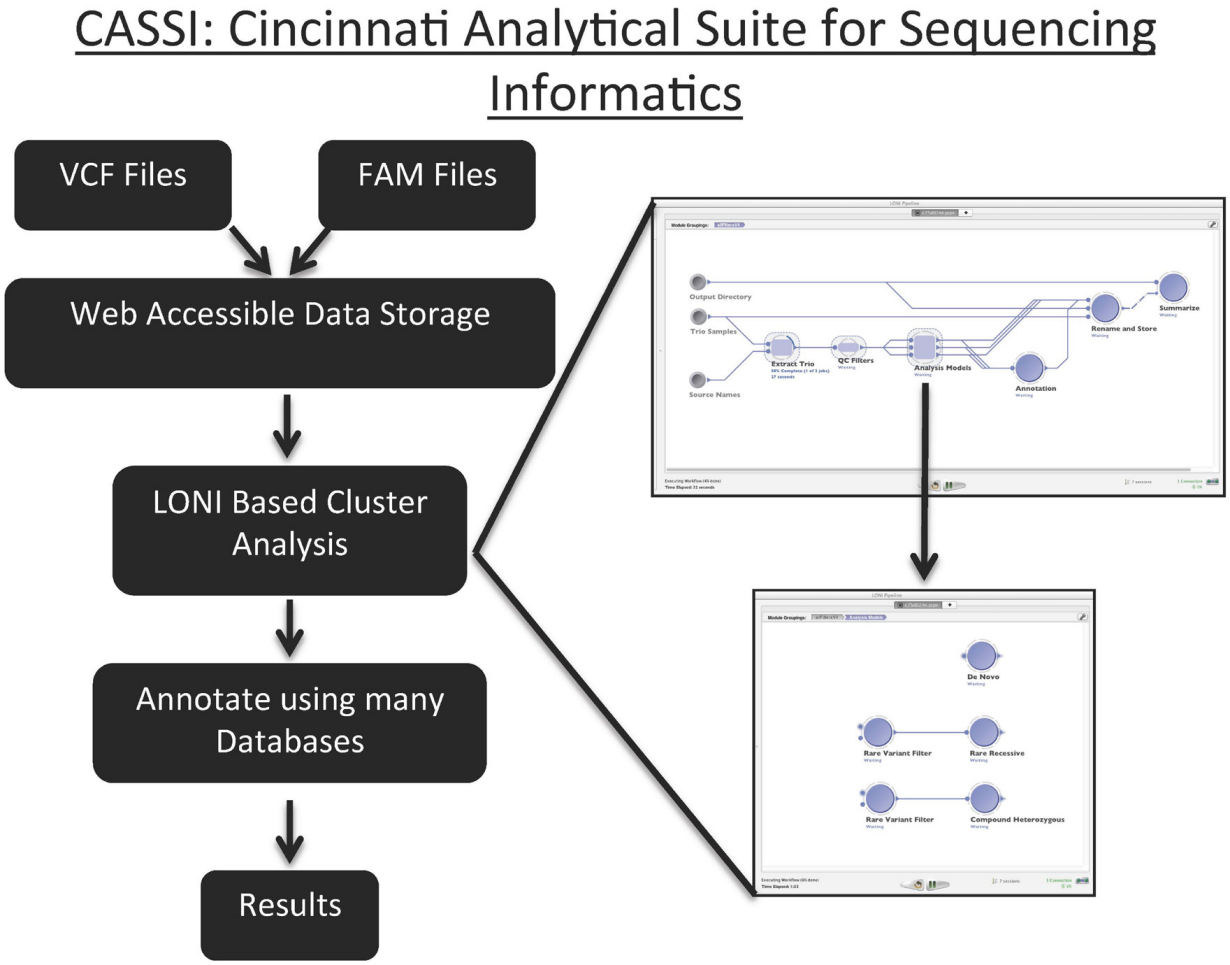
**Graphical Representation of the Cincinnati Analytical Suite for Sequencing Informatics.** Files with VCF and FAM are uploaded to CASSI’s web interface. These data are parsed and stored in web accessible data storage. The LONI based analysis allows users to analyze data through the established pipeline for identifying *de novo*, rare recessive and compound heterozygous variants. These pipe lines can also be changed to accommodate specific analyses. After variants are annotated using many databases, the results are versioned, saved in the database, and available for downloading.

We developed CASSI to meet the need to store, version, filter, and annotate NGS data. CASSI is an application that seamlessly integrates file storage, metadata storage (e.g., family structure), and downstream processing with a web-based front-end that contains a user-friendly query interface (**Figure 7**). The web interface of CASSI enables biologists and clinicians without any computer science background to launch sophisticated analytical workflows to analyze next-generation sequencing data in an automated procedure. For example, the interface allows users to directly interface with annotation and filtering packages (such as vcftools, variant tools, and ANNOVAR), which are executed on a high-performance cluster at CCHMC.

The key technical component in CASSI is the LONI pipeline engine from UCLA, which is a graphical user interface for executing complex workflows on a cluster that can be launched directly from a web browser. Query results obtained through the CASSI web interface are made available as a data source in the LONI pipeline, and users can choose from a large number of filtering and annotation workflows to analyze variant data. CASSI allows the user to efficiently compare various filtering strategies; for example, it can easily record the number of variants and Mendelian errors remaining after individual filters are implemented. Most importantly, CASSI can be used to assess concordance between samples and to identify *de novo*, rare recessive, and compound heterozygous variants. The flexibility of the pipeline facilitates the implementation of new analytical strategies directly from the interface. Other groups have independently developed a genomic pipeline using LONI, supporting the utility of this resource for sequencing data (Dinov et al., 2011; Torri et al., 2012; **Figure 6**).

## DISCUSSION

Next-generation sequencing provides investigators with the ability to quickly and economically generate human sequencing data including the presence of SNPs and insertions/deletions (O’Rawe et al., 2013). This ability to generate large volumes of data also presents the challenge of determining which variants to validate and study biologically (Nielsen et al., 2011). As reviewed in *Nekrutenko and Taylor*, there is no generally accepted method for filtering variants in clinical studies (Nekrutenko and Taylor, 2012). The usual approach for shortening the list of top variants relies on filtering on two parameters, read-depth, and PHRED quality score (Girard et al., 2011; Xu et al., 2011; O’Roak et al., 2012a; Sanders et al., 2012). Although these particular methods successfully remove many of the variants, due to the stringency of filters, they are also excluding real variants present within the sequencing data. By using a combination of three filters based on the intrinsic characteristics of NGS, we removed a large proportion of the Mendelian errors, while retaining the highest portion reasonable of variants called. We estimate that these filters removed 90% of the sequencing artifacts at a cost of 20% of the data.

One limitation of using Mendelian errors to identify sequencing artifacts is that they under represent the true sequencing error rate as they have low power to detect errors in bi-allelic polymorphisms. In cases where both parents are heterozygous for a polymorphism the child could have any one of the potential genotypes and it would follow Mendelian inheritance. In genotyping experiments it has been estimated that Mendelian errors only predict one-third to one-fourth the number of actual errors (Gordon et al., 1999). Furthermore, the identification of Mendelian errors does not indicate which sample’s genotype is erroneous. Even with the limitations, Mendelian errors provide a useful method to determine the quality of the data.

The number of variants with a particular depth of coverage demonstrated a clear peak around 120 (**Figure 1**), which was close to the target coverage depth of 100 reads. On the other hand, the histogram for the depth of coverage amongst Mendelian errors (**Figure 1**) confirmed that the majority of Mendelian errors had a low depth of coverage. This low depth of coverage for the Mendelian errors indicated that many of them may be occurring due to selective sequencing of one chromosome rather than equal sequencing of both chromosomes. This would be particularly relevant for heterozygous SNPs. If only one of the chromosomes was sequenced, the individual would be called either a homozygous reference or homozygous alternate at a particular variant. As the number of reads increases, the probability of sequencing the same chromosome for each read decreases exponentially. By sheer chance at a read depth of 10 with 50-million total reads, there will be 50,000 instances of only one chromosome being read. If the read-depth is increased to 15 reads, this number decreases to approximately 1,500 instances. Based on the difference in the distribution of Mendelian errors and total variants for depth of coverage, a filter which excludes variants with low depth of coverage (15 reads or <20% of average reads) removed a small portion of the total variants while removing a large portion of Mendelian errors (**Figure 2**).

The GQ is computed based on the likelihood of a particular genotype being called in comparison to the likelihood of the other two genotypes being called: L(0/1) versus L(0,0) and L(1,1). Our data based on the GQ demonstrated that the majority of variants have high GQ, whereas the Mendelian errors have a much lower score (**Figure 2**). The difference in the GQ represents the likelihood in the variant calls. For many of the Mendelian errors, the low GQ suggests a low confidence in those calls, which may be due to inconsistent individual sequencing reads at that particular location due to low coverage depth, difficult alignment, or poor sequencing reads. This is expected for sequencing artifacts, since it is unlikely that a sequencing artifact will consistently produce the same sequencing read at a particular location. On the other hand, a true Mendelian Error such as a *de novo* mutation would produce a consistent sequencing read since it is a true difference in the sequence. We exploited the differences in the genotype quality scores by generating a filter that excludes variants with a genotype quality score less than 20. This allowed us to exclude the Mendelian errors present on the left peak of the histogram without excluding a large portion of the called variants, which are located within the peak on the right side of the GQ histogram (**Figure 2**).

We added an additional filter based on the alternate allele ratio (alt ratio; DePristo et al., 2011; Girard et al., 2011; Xu et al., 2011; O’Roak et al., 2012a; Riviere et al., 2012; Sanders et al., 2012). Due to the high depth of coverage for most variants, we expected our variants to have alternate allele ratios close to the theoretical values: 0, 0.5, and 1 representing homozygous reference, heterozygous, and homozygous alternate, respectively. In effect, this filter assesses the consistency of the variant call based on all the sequencing reads. The majority of the variants that the alt ratio filter removes were heterozygous Mendelian errors which were enriched in the peak at 0.2 (*p*-value < 10^-^^50^; **Figure 3**) suggesting that homozygous reference and homozygous alternate variant calls were more reliable than heterozygous variant calls.

We combined these three individual filters and observed the increased efficiency of the combined filters in removing the sequencing artifacts. As is evident from the bar graph representing the total number of variants (**Figure 5**), and the line graph representing the number of Mendelian errors called, there was a 93% decrease, in the number of Mendelian errors by the addition of the GQ filter to the Alt Ratio without a large reduction in the number of variants removed (14.9%). This trend continued as we added the depth of read filter to the other filters. By excluding variants that fail the depth of read filter, the Genotype Quality Score Filter, or the Alt Ratio filter, we were able to exclude over 95% of the Mendelian errors. In our test trio, this lowered the number of Mendelian errors called to approximately 130 variants. We attributed the drastic decrease in the number of Mendelian errors to the low likelihood of a sequencing artifact passing all the filters. Approximately 80% of the variants passed all three filters.

After identifying a combination of filters that removed the vast majority of the Mendelian errors, while retaining a large portion of the variants called, we assessed the concordance between identical samples isolated from different DNA sources. The unfiltered sequencing data-set of samples from blood, buccal cells, and saliva had a concordance of ~84% (including unique calls as discordant). After applying our filters the concordance rate increased to >99.9% between all three samples from the three different DNA sources. It is necessary for the filtering method to generate concordant data, since clinical DNA samples can be collected from any one of various different sources including blood, saliva, and buccal cells.

Next-generation sequencing experiments are often used to find rare or novel variants that lead to disease. Unfortunately, sequencing artifacts can often mimic and confound the identification of these variants. Sequencing artifacts contribute to a large number of false positive disease-causing candidates. The vast majority of apparent *de novo* variants identified in the unfiltered data are sequencing artifacts (**Table 2**). However in previous experiments, after filtering the data on read depth, genotype quality score and alt ratio our confirmation rate by Sanger sequencing is greater than 95% for *de novo* variants.

Cincinnati Analytical Suite for Sequencing Informatics is a suite that allows users with varying degrees of programming sophistication to perform documented, reproducible studies with NGS data to gain insight into the etiology of disease. In the case of the current study, CASSI allowed us to quickly and reproducibly assess different filtering strategies through the calculation of Mendelian errors and total variants remaining after specific filters were applied. With the incorporation of a LONI pipeline we have created a fully automated system that can filter, annotate and apply various genetic models to identify candidate causative variants. The LONI pipeline provides investigators the ability to apply predefined values for filtering or customize the pipeline to fit the type and quality of the data being analyzed.

The filters and methods presented reproducibly generate robust and accurate data sets with low levels of sequencing artifacts. Both the genotype quality score and alt allele ratio filters can be applied to data sets regardless of read depth. In data sets with >75× average read depth we recommend using a hard filter of 15× for read depth. In data sets with less than 75× coverage we suggest using a filter of 20% of the average read depth. While these data sets will contain higher amounts of false variant calls, a hard filter would remove too many true variants from the data set. These filters also have the potential to have bias towards removing variants caused by mosaicism. The alt allele ratio would be particularly sensitive to variants if the cell population with different genotypes is not close to 50%.

As the NGS technology progresses further and the per-base sequencing cost decrease, researchers will be able to generate NGS data-sets with increased depth of coverage and longer read lengths. Both of these improvements will yield better calling of variants. Additionally, the longer read lengths will allow researchers to more accurately predict insertions and deletions. A recent review further identifies ways to improve the fidelity of NGS data, including the use of filtering strategies such as the one presented herein (Robasky et al., 2014).

In summary, our three filters of NGS data selectively exclude the sequencing artifacts, measured as Mendelian errors, while limiting the removal of the true variation amongst the samples. In addition, we show that DNA isolated from different sources (blood, buccal cells, and saliva) have greater than 99.9% concordance and thus mixed DNA sources can be used for causative variant identification. Our work flow is based on obtaining the most accurate data set possible and results in an extremely small number of candidate causative variants for consideration and interpretation (usually fewer than 10 genes per trio). These methods have been automated through CASSI and greatly increase the ability of investigators and clinicians to understand and discover genetic causes of disease by quickly identifying potential causative variations.

## Conflict of Interest Statement

The authors declare that the research was conducted in the absence of any commercial or financial relationships that could be construed as a potential conflict of interest.

## Footnotes

1 http://hgdownload.soe.ucsc.edu/goldenPath/hg19/bigZips/

2 ftp://ftp-trace.ncbi.nih.gov/1000genomes/ftp/

3 http://evs.gs.washington.edu/EVS/
